# Singing for lung health in COPD: a multicentre randomised controlled trial of online delivery

**DOI:** 10.1136/bmjresp-2024-002365

**Published:** 2024-05-02

**Authors:** Keir E J Philip, Sara C Buttery, Sarah Bowen, Adam Lewis, Edmund Jeffery, Saeed M Alghamdi, Parris Williams, Ali M Alasmari, Abdullah S Alsulayyim, Christopher M Orton, Francesca Conway, Ley Chan, Bavithra Vijayakumar, Anand Tana, James Tonkin, Alexis Perkins, Justin L Garner, Karthikan Srikanthan, Ahmed Sadaka, Matthew J Pavitt, Winston Banya, Adam Lound, Sarah Elkin, Michael I Polkey, William D-C Man, Keir Lewis, Phoene Cave, Daisy Fancourt, Nicholas S Hopkinson

**Affiliations:** 1 National Heart and Lung Institute, Imperial College London, London, UK; 2 Respiratory Medicine, Royal Brompton and Harefield Hospitals, London, UK; 3 Hywel Dda University Health Board, Carmarthen, UK; 4 Department of Health Sciences, Brunel University London, Uxbridge, UK; 5 Clinical Technology Department, Faculty of Applied Medical Sciences, Umm Al-Qura University College of Applied Medical Science, Makkah, Saudi Arabia; 6 Department of Respiratory Therapy, College of Medical Rehabilitation, Taibah University, Madinah, Al Madinah, Saudi Arabia; 7 Respiratory Therapy Department, Faculty of Applied Medical Sciences, Jazan University, Jazan, Saudi Arabia; 8 Department of Chest Diseases, Alexandria University Faculty of Medicine, Alexandria, Egypt; 9 Patient Experience Research Centre, Imperial College, London, UK; 10 Respiratory Medicine, Imperial College Healthcare NHS Trust, London, UK; 11 Department of Behavioural Science and Health, University College London, London, London, UK

**Keywords:** Pulmonary Rehabilitation, Respiratory Muscles, Exercise, Emphysema, Complementary Medicine

## Abstract

**Background:**

Singing for lung health (SLH) is an arts-based breathing control and movement intervention for people with long-term respiratory conditions, intended to improve symptoms and quality of life. Online, remotely delivered programmes might improve accessibility; however, no previous studies have assessed the effectiveness of this approach.

**Methods:**

We conducted an assessor-blind randomised controlled trial comparing the impact of 12 weeks of once-weekly online SLH sessions against usual care on health-related quality of life, assessed using the RAND 36-Item Short Form Health Survey (SF-36) Mental Health Composite (MHC) and Physical Health Composite (PHC) scores.

**Results:**

We enrolled 115 people with stable chronic obstructive pulmonary disease (COPD), median (IQR) age 69 (62–74), 56.5% females, 80% prior pulmonary rehabilitation, Medical Research Council dyspnoea scale 4 (3–4), forced expiratory volume in 1 s % predicted 49 (35–63). 50 participants in each arm completed the study. The intervention arm experienced improvements in physical but not mental health components of RAND SF-36; PHC (regression coefficient (95% CI): 1.77 (95% CI 0.11 to 3.44); p=0.037), but not MHC (0.86 (95% CI −1.68 to 3.40); p=0.504). A prespecified responder analysis based on achieving a 10% improvement from baseline demonstrated a response rate for PHC of 32% in the SLH arm and 12.7% for usual care (p=0.024). A between-group difference in responder rate was not found in relation to the MHC (19.3% vs 25.9%; p=0.403).

**Discussion and conclusion:**

A 12-week online SLH programme can improve the physical component of quality of life for people with COPD, but the overall effect is relatively modest compared with the impact seen in research using face-to-face group sessions. Further work on the content, duration and dose of online interventions may be useful.

**Trial registration number:**

NCT04034212.

**WHAT IS ALREADY KNOWN ON THIS TOPIC**

**WHAT THIS STUDY ADDS**

- Online singing for lung health produced statistically significant improvements in the physical component of health-related quality of life, but the mean effect size was small.

**HOW THIS MIGHT AFFECT RESEARCH, PRACTICE OR POLICY**

- Although singing for lung health can be delivered online, the effects are modest. Further research should focus on face-to-face delivery or consider if or how online interventions could be made more effective (content, dose and duration).

## Introduction

 Chronic obstructive pulmonary disease (COPD) is a common, progressive, long-term respiratory condition characterised by persistent airflow limitation and symptoms, including dyspnoea, cough and exercise limitation, with further impacts including depression, anxiety, social isolation and loneliness, all contributing to reduced quality of life.^1^,^6^

Singing-based interventions, incorporating elements of breathing control and physical activity, have been identified as potentially useful for breathless people with chronic respiratory diseases, including COPD.^7^,^12^ Participants in singing for lung health (SLH) programmes report high levels of enjoyment, symptom improvement and improved quality of life.^7 8 13^ A Cochrane systematic review from 2017 found low to very low-quality evidence suggesting that SLH could improve physical health and called for longer term, adequately powered studies to allow greater confidence regarding its effect.^14^ Most recently, one large randomised controlled trial (RCT) suggested that SLH could have non-inferior impacts on physical performance (assessed with the 6-minute walk test (6MWT)) when substituted for the physical training component of pulmonary rehabilitation (PR) for people with COPD,^11^ and a 2002 systematic review of the effect of singing in COPD demonstrated improvements in quality of life (36-Item Short Form Health Survey (SF36) Physical Health Composite (PHC)) and respiratory muscle strength.^15^ Interest in developing digitally delivered interventions, including singing-based interventions, has been increased by the COVID-19 pandemic and related public health measures.^1216^,^18^

The primary aim of this study was to test the hypothesis that participation in SLH would improve health-related quality of life in people with COPD, with secondary measures of breathlessness, disease impact on quality of life, anxiety, depression, balance, confidence and physical activity. The initial trial registration was planned to investigate participation in face-to-face SLH, but this was amended due to the COVID-19 pandemic, to instead investigate the effect of an online programme.

## Methods

Singing for Health: Improving Experiences of Lung Disease (SHIELD) Online Trial was a multicentre, multinational, single-blind, RCT of online SLH compared with usual care (UC) for people with COPD. The study was originally conceived to investigate the impact of face-to-face SLH but was adapted to online delivery due to the COVID-19 pandemic.^13^ The study was conducted in accordance with the Declaration of Helsinki, received ethical approval from the National Health Service Health Research Authority, Stanmore REC (19/LO/0418) and was prospectively registered at Clinicaltrials.gov (NCT04034212) where the protocol is also available. We used the Consolidated Standards of Reporting Trials checklist when writing our report.^19^

### Study participants

**Figure 1:**
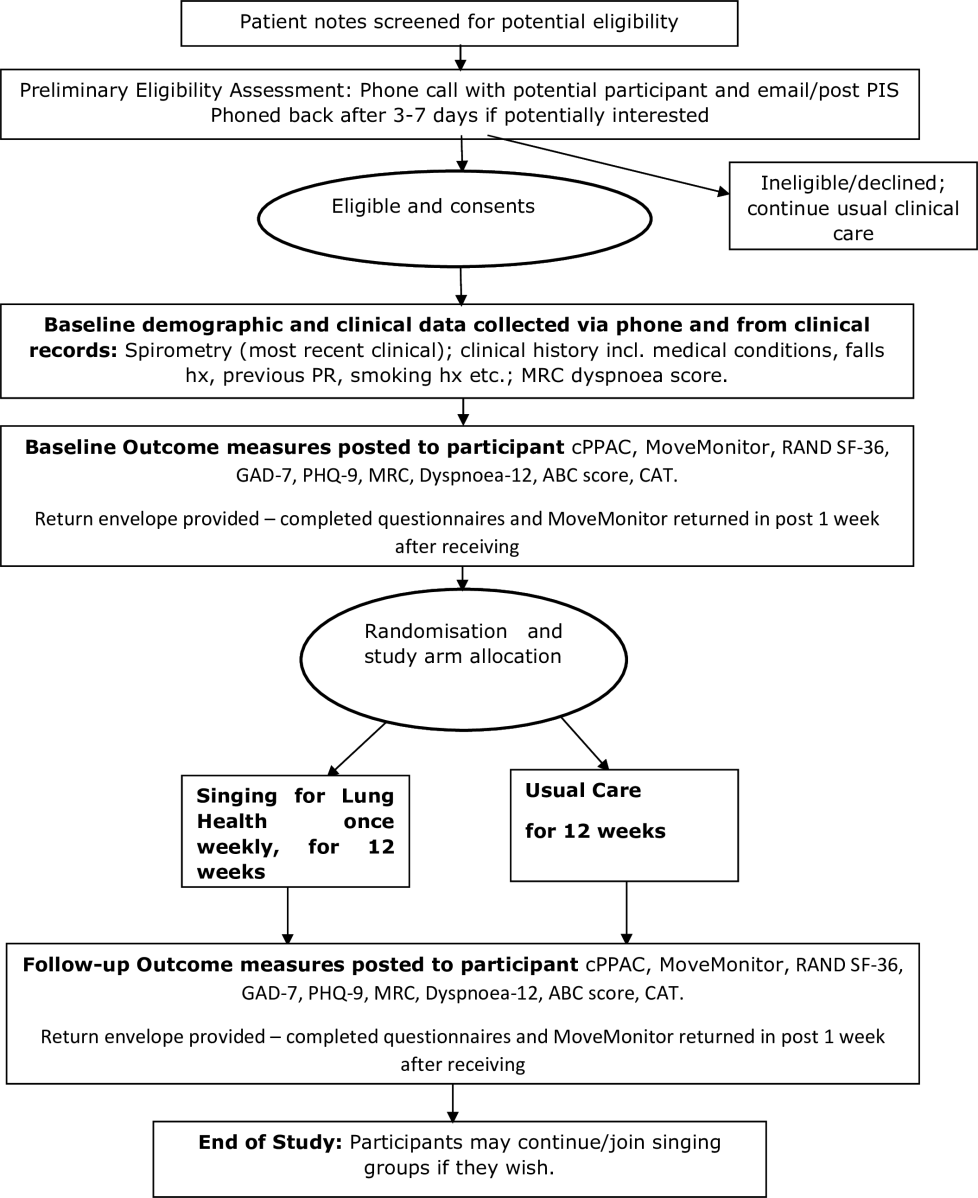
Singing for Health: Improving Experiences of Lung Disease trial study procedures.CAT, chronic obstructive pulmonary disease assessment test; GAD-7, Generalised Anxiety Disorder-7 questionnaire; MHC, Mental Health Composite; MRC, Medical Research Council; PHC, Physical Health Composite; PHQ-9, Patient Health Questionnaire (9 question); SF-36, 36-Item Short Form Health Survey.

Participant flow through the study is shown in figure 1. The inclusion criteria were adults with stable COPD. Potential participants were excluded if they had current or recent (within 4 months) participation in pulmonary rehabilitation, unable to take part in singing sessions due to comorbidity (eg, life-limiting illness and cognitive impairment) and had previously participated in SLH classes. Additionally, following the pandemic, which necessitated adaptation from face-to-face to online delivery, potential participants were also required to have internet access with a suitable device (computer, tablet or phone) capable of accessing video conferencing applications.

### Study interventions

SLH has been developed with the intention of improving symptoms in people with long-term respiratory conditions using singing techniques. Training programmes for singing leaders and the way that SLH is delivered have been evaluated to confirm intervention fidelity.^20^ The intervention arm participated in an online SLH programme. This included an initial one-to-one phone call between the session leader and each participant, 12 once-weekly online SLH group sessions (each lasting 1 hour) and online resources to support the sessions, which can be accessed here: https://www.youtube.com/playlist?list=PLNwZhuPSkc4UUOE-9IAEgWJpknhZlBGkL. Session content was tailored to participants’ respiratory condition, severity and type of symptoms experienced, prior singing experience, confidence and capabilities, and demographic considerations, including avoiding music with religious or potentially offensive content. The content was adapted iteratively, responding to participant feedback, progress and session-to-session variations in mood and health. For example, if the group was feeling particularly energetic, an extra repetition of an exercise might take place, or if the participants said they were feeling a bit tired, they would be invited to take extra breaks and join in when they wanted. Being small and highly variable in nature, these adaptations were not formally monitored. No major adaptations to the content took place. Repertoire selection was done in collaboration with session participants to ensure suitability and to increase the sense of contribution and control for participants. However, fidelity was maintained regarding the core components, duration and methods of delivery.^9^ The full components are outlined in table 1.

**Table 1:** Overview of the SLH online programme

Component	People present	Description	Content
One-to-one session	SLH session leader and participant	Telephone call to trouble shoot potential technical difficulties (20 min)	Opportunity to ask questions about SLH programme content. Discuss suitability for the programme at current time if anything might have changed, including clinical status and timings of sessions. Address potential access issues related to technical connection issues. Build trust with less confident participants.
Weekly online group sessions	SLH session leader specialist with up to 15 participants	Online Group SLH sessions (twelve 1-hour sessions).	Breathing and mindfulness exercise to bring their mental focus to their body. Physical warm-up exercises to stretch muscles ready for singing, increase use of coordination exercises with words and sound to engage participants. Vocal warm-ups and exercises, including vocal fricatives, rhythm games, singing and movement exercises to help overcome holding patterns attached to their breath or bad habits, for example, lightly rolling shoulders backwards during vocal exercise or the line of a learnt song. Exercises using primal sounds (sigh, whinge and sirens to different vowel sounds) to explore vocal placement and settings. Song repertoire. Songs are selected based on difficulty, adaptability to include medium to long phrase lengths, further practicing techniques learnt through vocal warmups. Regular points of discussion, exploring experience of content, difficulty level/effort level after adding movement or changing technique. Emphasis on combining gentle movement with. Summary of content covered. A wide range of repertoire from world folk music to well-known popular songs, some requested by participants to encourage more engagement in the sessions. In simpler songs, singing harmony and round singing is was explored. At the end of sessions, participants were encouraged to use the online videos.
Online resources	Participant self-directed	Bespoke online digital resources to support participants between sessions (as per participant’s preference)	Videos (and audio recordings) of vocal exercises and songs used in the sessions. Backing tracks so they could sing with the learnt songs through the week. Further exercises and tools for daily practice or as frequently as the participant feels is appropriate for them. Examples of online video resources (https://www.youtube.com/playlist?list=PLNwZhuPSkc4UUOE-9IAEgWJpknhZlBGkL)
Regular emails and point of contact provided	All participants during the 12-week course	Emails from group leaders to participants	Presession email to remind participants to attend upcoming session Email following session: during the week rather than straight after to encourage practice mid-week) summarises session content and remind participants of key exercises that can be practiced during the week with links to online resources. Other (one-to-one) emails, throughout the week, depending on the needs of each participant, with the primary intention of supporting attendance and identifying any potential issues arising. All emails received from participants were answered by the SLH session leader. SLH leaders are supported by clinical members of the research team for any clinical questions, queries and for reporting adverse events.

SLH, Singing for Lung Health.

The control arm continued with UC, as directed by their usual medical team, and did not receive any additional intervention beyond UC as part of the study.

After exiting the study, the UC arm participants were offered a place in an online SLH 12-week course. The SLH intervention arm also continued with their UC in addition to the SLH intervention.

### Study outcomes

The primary outcome was a change in health-related quality of life (HRQoL), assessed using the RAND SF-36 tool Mental Health Composite (MHC) and PHC scores, comparing SLH to UC. Secondary outcome measures were changes in the COPD assessment test (CAT), Generalised Anxiety Disorder Assessment (GAD-7), Patient Health Questionnaire-9 (PHQ-9), Dyspnoea-12 questionnaire (D12), Activities-specific Balance Confidence scale, PROactive cPPAC tool and daily step count. The daily step count was calculated by the mean number of steps taken calculated from ‘valid days’ (≥8 hours of wearing time) by dividing the total steps from valid days by the number of valid days collected, with a minimum of four valid days data required, as per validation studies for the devices used.^21 22^

The move to online delivery during the pandemic meant that we did not include some of the secondary endpoints specified in our initial trial registration that would have required an in-person assessment, namely the 6MWT and the Short Physical Performance Battery (SPPB). Removal of these outcome measures was prospectively described in an amendment to the ClinicalTrials.gov record (NCT04034212).

The most recent clinically assessed spirometry was used as non-essential spirometry was not being conducted at our institution due to the COVID-19 pandemic. Baseline demographics and clinical information were collected by phone. Patient public involvement consultation when developing the study protocol suggested posting the questionnaires was preferred to using an online form. When questionnaires were not returned adequately completed, two attempts were made to call the participant. In certain cases, it was not possible to collect sufficient responses for a questionnaire, and as such, the denominator varies slightly between outcome measures, with missing data described in the results. Physical activity data were collected by posting the MoveMonitor to participants. The device is worn in the middle of the back on an elasticated belt around the waist, for a full week, day and night, only being removed if having a bath or shower.^23 24^ Instructions were provided over the phone with the opportunity to ask questions, with further step-by-step instructions sent in writing with the device.

All outcome measures were assessed at baseline prior to randomisation and again after the intervention period. Clinical support for the SLH session leader was provided by clinical team members, with participant study arm allocation masked or unmasked as appropriate. Participants were randomised (1:1) using computer-generated randomisation lists (Sealed Envelope) block size 4, stratified by Medical Research Council (MRC) breathlessness level and previous participation in pulmonary rehabilitation.

Study participants were encouraged to report any illness, or other potential adverse event, to the study team, with contact details provided. The SLH session leader also regularly inquired with participants regarding potential adverse events, and reasons for non-attendance were sought. Finally, an adverse event reporting form was included in the follow-up data collection to provide an additional opportunity to catch any potential issues that had not been identified previously.

### Sample size and statistical analysis

We powered the study to have a 90% chance to detect a 10-point change in RAND SF-36 MHC and PHC, assuming, based on previous work by our group,^7 8^ a SD for change in SF-36 of 15 points. At a 5% significance level, 48 patients would be required in each arm. Allowing for a 20% loss to follow-up, the recruitment target was 120 participants.

The primary analysis was on an intention-to-treat (ITT) basis. Between-group change in outcome measures was compared using linear regression, including the baseline level of the outcome of interest as a covariate. The regression coefficients correspond to an estimate of the effect size related to intervention participation. A prespecified responder analysis comparing the percentage in each arm achieving a 10% improvement in PHC and MHC scores was also conducted. Previous SLH in COPD studies demonstrated baseline PHC and MHC scores of 30–50,^7 8^ and therefore, a 10% change was selected for the responder analysis as this corresponds to the PHC/MHC minimal clinically important difference (MCID) of 3–5 points, which is typically used in other conditions.^25^ Missing data have been imputed using the last/baseline recording carried forward method, for 15 (13%) participants, 7 and 8 participants for intervention and UC arms, respectively. If baseline measures were missing, the measure was not included in the analysis. Complete case analyses are also presented in online supplemental file 1.

The normality of the distribution was checked using histograms. Data are presented as mean (SD), median (IQR) or number (%) as appropriate. Pre, post and change in outcomes are presented to aid interpretation. A p value of <0.05 was taken to indicate statistical significance. Statistical analysis was completed using STATA (V.15.1).

### Patient and public involvement

An expert patient research group at the Imperial College Biomedical Research Centre and existing SLH participants were directly involved in the design and conduct of this research. Specifically, they contributed to outcome measure selection and data collection methods.

## Results

**Figure 2:**
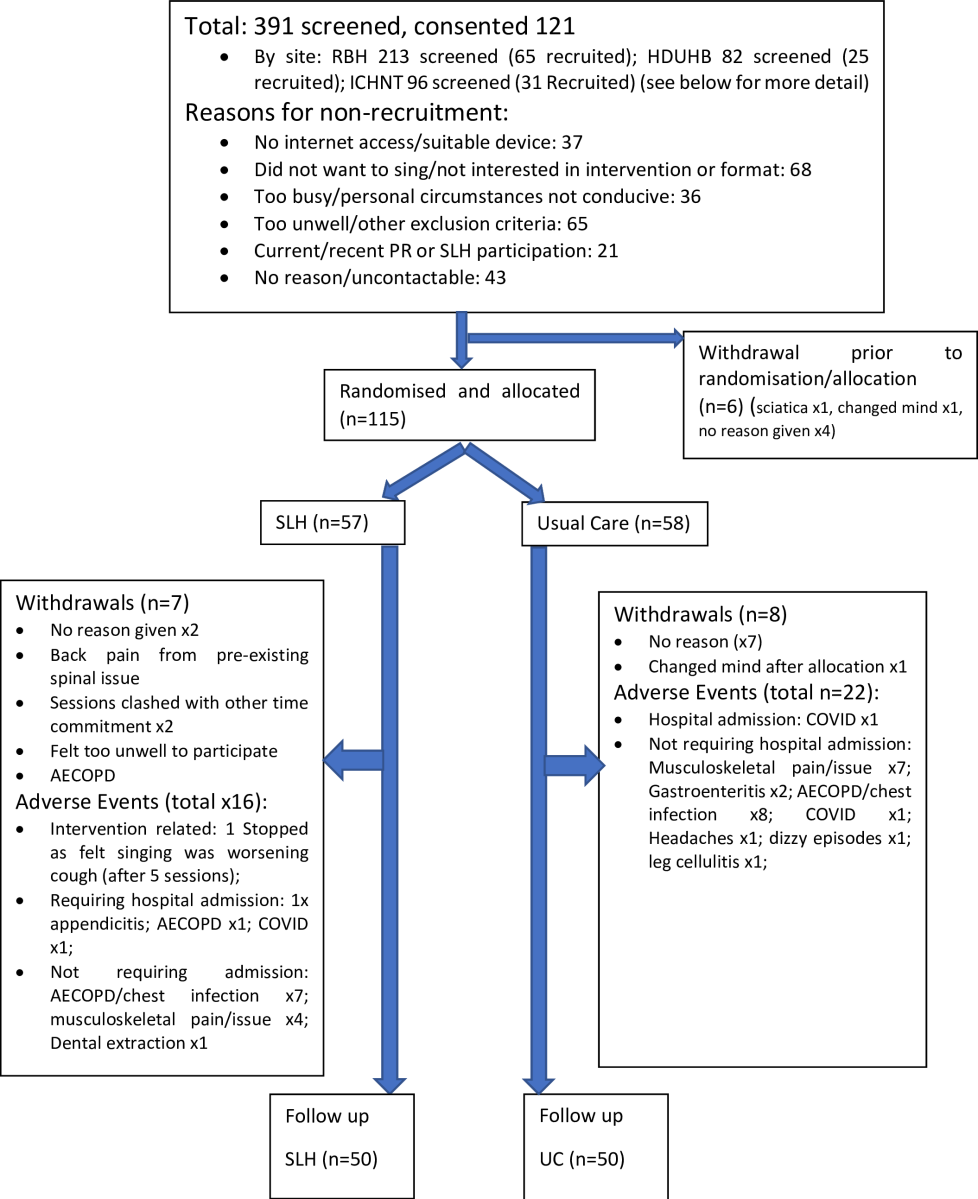
CONSORT diagram. CONSORT participant flow. AECOPD, acute exacerbation of chronic obstructive pulmonary disease; CONSORT, Consolidated Standards of Reporting Trials; SLH, Singing for Lung Health.

Participant recruitment took place between 14 October 2020 and 15 September 2021 at the Royal Brompton Hospital, Imperial College Healthcare NHS Trust in London, England, and Hywel Dda University Health Board in Carmarthen, Wales. Participant’s recruitment and flow are shown in figure 2.

**Figure 3:**
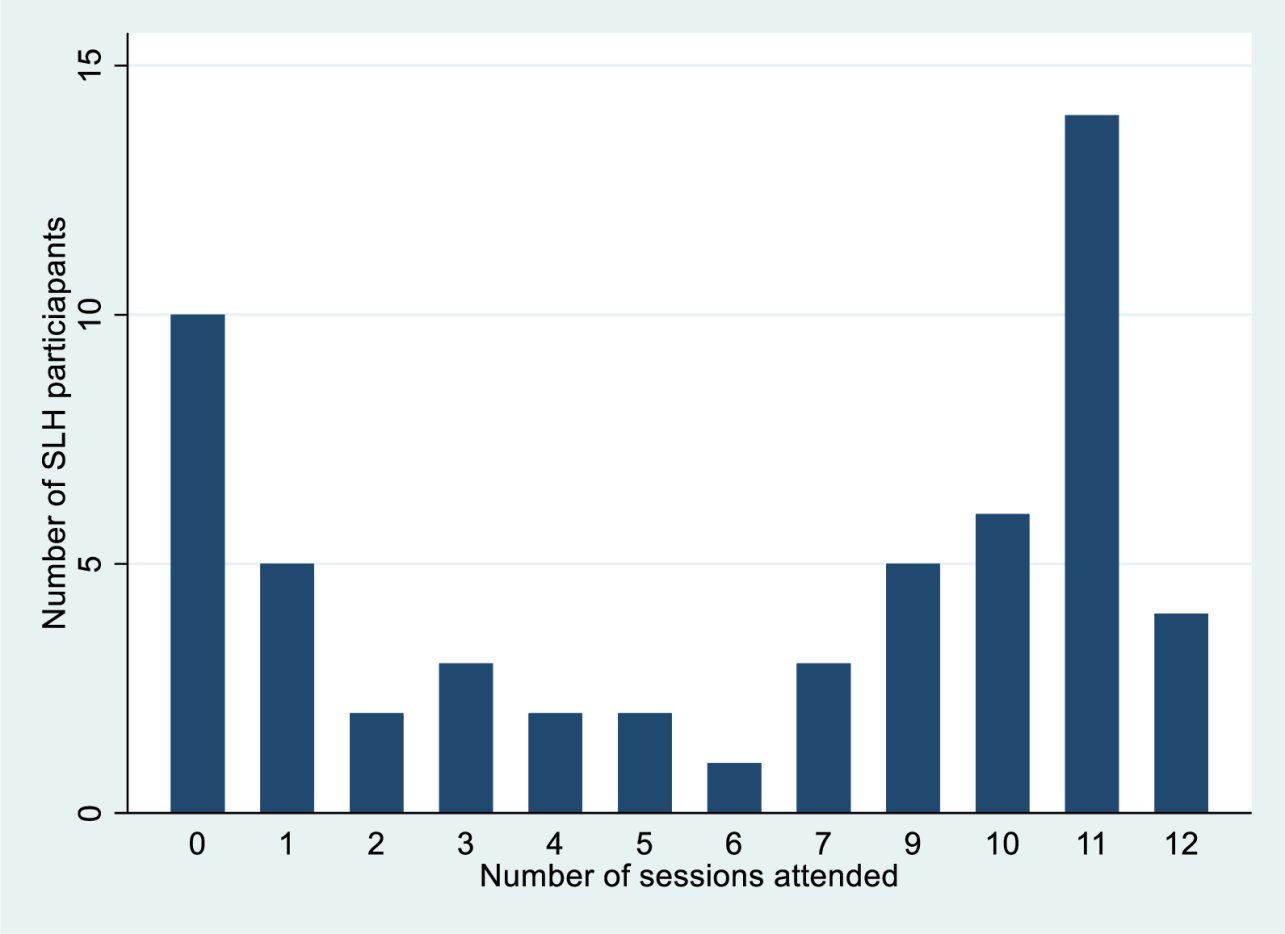
Singing for Lung Health session attendance.

The total study population, randomised and allocated to a group (n=115), had a median (IQR) age of 69 (62–74), were 56.5% females, 87.8% White British ethnicity, 11.3% from minority ethnic groups and 0.9% ethnicity not provided. 13% were current smokers, 80% previously participated in pulmonary rehabilitation, MRC median (IQR) 4 (3–4), forced expiratory volume in 1 s (FEV_1_) % predicted median (IQR) 49 (35–63), and 10.4% were using supplementary oxygen therapy. The study arms were well matched at baseline in terms of demographic, clinical and outcome measures (table 2). Median attendance at the 12 group sessions was 9 (IQR 1–11) for the intervention arm participants. A number of sessions attended in the intervention arm showed a ‘U’-shaped distribution (figure 3).

**Table 2:** Singing for Health: Improving Experiences of Lung Disease online baseline characteristics

	Intervention arm (n=57)	Usual care (n=58)
Age (median (IQR))	69 (62–73)	69 (63–74)
Gender (n (%))		
Female	32 (56.1%)	33 (56.9%)
Male	25 (43.9%)	25 (43.1%)
Other gender/prefer not to say	0	0
Ethnicity (n (%))		
White British	51 (89.5%)	50 (86.2%)
Minority ethnic group	6 (10.5%)	7 (12.1%)
Ethnicity not provided/stated	0 (0%)	1 (1.7%)
Number of comorbidities (median (IQR))	2 (1–2)	2 (1–2)
Smoking status		
Current	5 (8.8%)	10 (17.2%)
Ex-smoker	49 (86.0%)	44 (75.9%)
Never smoker	3 (5.3%)	4 (6.9%)
Pack years smoking (for current/ex-smokers) (mean (SD))	30.5 (16.6)	36.5 (23.2)
Previously participation in pulmonary rehabilitation	46 (80.7%)	46 (79.3%)
Falls in last year	0 (0–0)	0 (0–0)
Body mass index (mean (SD))	25.4 (5.4)	25.9 (7.2)
MRC dyspnoea score	3 (3–4)	3 (3–4)
Using supplementary oxygen	7 (12.3%)	5 (8.6%)
FEV_1_% predicted	48 (33.5–64.0)	49 (36–63)
**Outcome measures at baseline**		
RAND SF-36 PHC score	30.00 (22.4–36.4)	28.4 (22.3–35.8)
RAND SF-36 MHC score	36.7 (31.5–51.3)	36.4 (27.8 to 47.9)
SF-36 physical function	20.0 (5–40)	20.0 (10–45)
SF-36 role limitation, physical	0.0 (0.–50)	0.0 (0–50)
SF-36 pain	61.0 (22.0–84.0)	41.0 (22.0–62.0)
SF36 general health	25.0 (10–37)	21.0 (15–40)
SF-36 energy	35.0 (25–50)	25.0 (15.–45.)
SF-36 role limitation, emotional	66.7 (0–100)	33.33 (0–100)
SF-36 emotional well-being	68.0 (56–84)	60.0 (52–84)
SF-36 social functioning	50.0 (25–75)	50.0 (25–75)
CAT score (mean (SD))	23.5 (7.7)	23.1 (7.5)
Dyspnoea 12 (mean (SD))	17.7 (9.3)	18.7 (9.1)
MRC dyspnoea score	4 (3–4)	4 (3–4)
Depression (PHQ-9)	7.0 (2.0–12)	10.0 (3–14)
Anxiety (GAD-7)	2.5 (0–7.5)	4.0 (1–7)
ABC score	75.6 (40–88.1)	73.13 (48.1–89.7)
PROactive total score (mean (SD))	50.5 (16.2)	52.7 (14.2)
PROactive amount score (mean (SD))	45.2 (21.6)	45.7 (19.7)
PROactive difficulty score (mean (SD))	56.0 (16.8)	57.9 (15.5)
Daily step count	3678 (2191–5414)	3576 (2174– 4568)

Data shown are median (IQR), or if appropriate mean (SD)/number (%) as indicated.

ABC, Activities-specific Balance Confidence; CAT, chronic obstructive pulmonary disorder assessment test; FEV_1_, forced expiratory volume in 1 s; GAD-7, Generalised Anxiety Disorder-7 questionnaire; MHC, Mental Health Composite; MRC, Medical Research Council; PHC, Physical Health Composite; PHQ-9, Patient Health Questionnaire (9 question); SF-36, 36-Item Short Form Health Survey.

**Figure 4:**
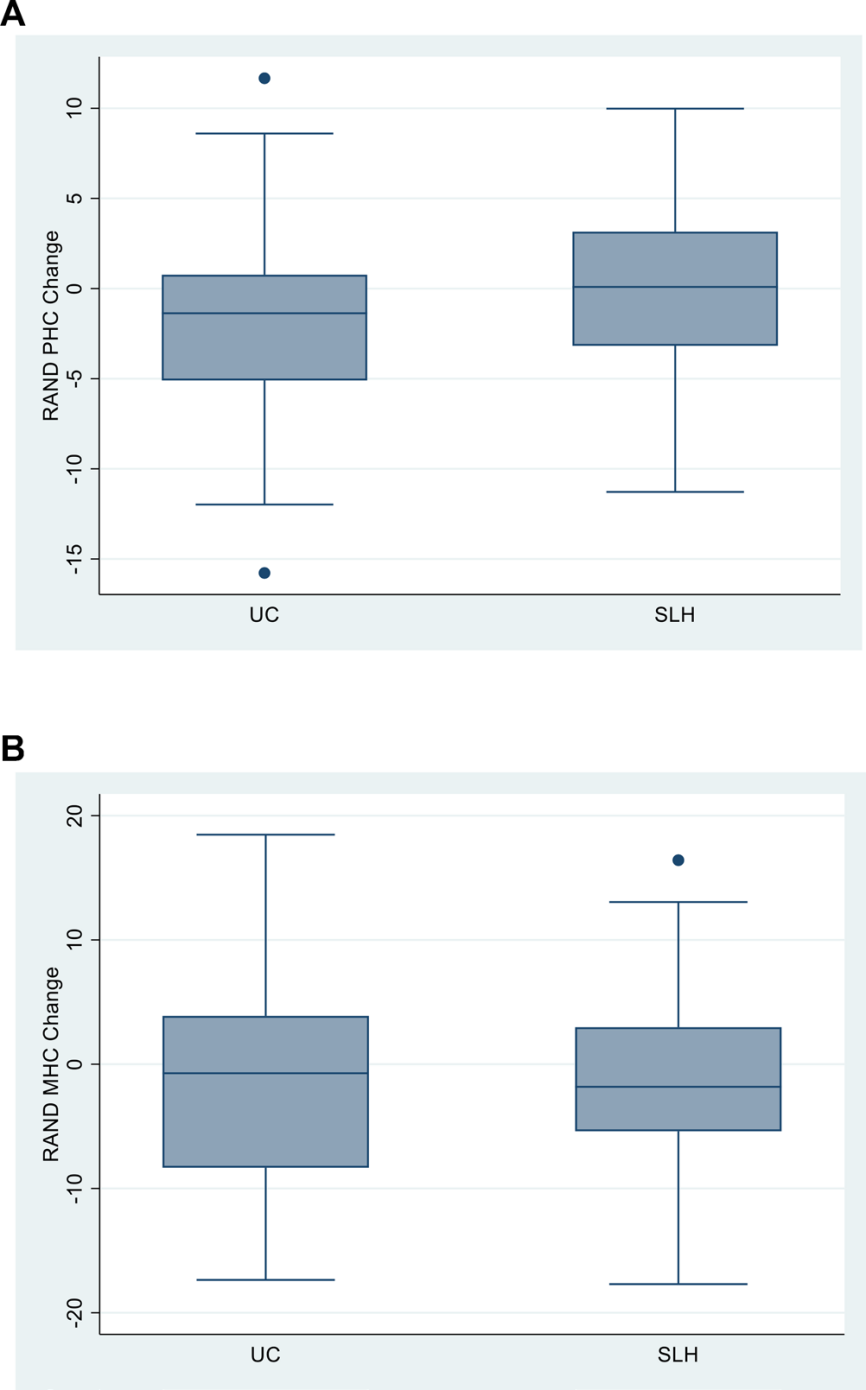
Impact of Singing for Lung Health on health-related quality of life. MHC mental health component score of RAND SF-36; PHC physical health component score. Boxes indicate 25th to 75th percentile; central line is the median; whiskers are upper and lower adjacent values; outliers are values beyond the upper and lower adjacent values. MHC, Mental Health Composite; PHC, Physical Health Composite; SF-36, 36-Item Short Form Health Survey.

The SF-36 PHC score improved in the SLH arm compared with usual care (regression coefficient +1.77 (95%CI 0.11 to 3.44); p=0.037), but the change in SF-36 MHC did not differ between groups (+0.86 (95%CI −1.68 to 3.40); p=0.504) (figure 4, and table 3). The responder analysis based on achieving a 10% improvement in SF-36 PHC from baseline showed a statistically significant difference between study arms of 28.1% for SLH versus 11.1% for UC (p=0.025) but did not differ for the SF-36 MHC (19.3% SLH vs 25.9% UC, p=0.403), giving a number needed to treat (NNT) of six. A similar treatment effect was seen in the complete case responder analysis, with 32% of the SLH arm and 12.7% of the UC (p=0.024) experiencing a 10% increase from baseline in SF-36 PHC, but not for the SF-36 MHC (SLH 22% vs UC 29.8%, p=0.381) (table 4). Secondary outcome measures did not meet the predefined level of significance of p<0.05.

**Table 3:** Comparison of outcomes between study arms, intention-to-treat (imputation of missing data using last carried forward)

	Singing for Lung Health online (n=57)			Usual care (UC) (n=58)				
Outcome variable	Baseline	Follow-up	Change (mean (SD))	Baseline	Follow-up	Change (mean (SD))	Regression coefficient (95% CI)	P value
RAND SF-36 PHC score	30.00 (22.43–36.38) (n=57)	29.79 (23.39–35.50)	−0.12 (4.93)	28.37 (22.32–35.77) (n=54)	26.71 (21.33–33.72)	−1.83 (4.77)	1.77 (0.11 to 3.44)	0.037
RAND SF-36 MHC score	36.71 (31.50–51.28) (n=57)	39.57 (29.73–48.15)	−0.96 (6.74)	36.44 (27.76–47.89) (n=54)	36.00 (28.06–42.04)	−1.31 (7.58)	0.86 (−1.68 to 3.40)	0.504
SF-36 physical function	20.00 (5.00–40.00)	25.00 (10.00–45.00)	2.98 (13.66)	20.00 (10.00–45.00)	25.00 (10.00–45.00)	0.09 (11.51)	2.43 (−2.12 to 6.99)	0.292
SF-36 role limitation, physical	0.00 (0.00–50.00)	0.00 (0.00–50.00)	−2.19 (26.42)	0.00 (0.00–50.00)	0.00 (0.00–25.00)	−11.57 (30.99)	8.81 (−0.55 to 18.16)	0.065
SF-36 pain	61.00 (22.00–84.00)	52.00 (31.00–84.00)	−0.65 (24.02)	41.00 (22.00–62.00)	41.00 (22.00–62.00)	−2.98 (18.70)	4.78 (−2.70 to 12.27)	0.208
SF-36 general health	25.00 (10.00–37.00)	20.00 (10.00–35.00)	−1.19 (12.20)	21.00 (15.00–40.00)	20.00 (10.00–35.00)	−2.50 (11.24)	1.03 (−3.19 to 5.25)	0.629
SF-36 energy	35.00 (25.00–50.00)	35.00 (25.00–50.00)	−0.26 (14.22)	25.00 (15.00–45.00)	32.50 (15.00–45.00)	0.09 (14.68)	1.50 (−3.43 to 6.44)	0.547
SF-36 role limitation, emotional	66.67 (0.00–100.00)	66.67 (0.00–100.00)	2.34 (39.27)	33.33 (0.00–100.00)	33.33 (0.00–100.00)	−3.09 (44.52)	7.53 (−6.60 to 21.67)	0.293
SF-36 emotional well-being	68.00 (56.00–84.00)	68.00 (52.00–80.00)	−1.82 (12.94)	60.00 (52.00–84.00)	62.00 (40.00–76.00)	−2.81 (14.94)	2.07 (−2.87 to 7.01)	0.407
SF-36 social functioning	50.00 (25.00–75.00)	50.00 (25.00–75.00)	−4.39 (20.11)	50.00 (25.00–75.00)	43.75 (25.00–62.50)	−3.47 (27.41)	−0.39 (−8.52 to 7.73)	0.924
CAT score	25.00 (18.00–29.00) (n=57)	24.00 (17.00–29.00)	−0.18 (4.43)	24.50 (16.00–29.00) (n=54)	24.00 (16.00–31.00)	0.67 (5.03)	−0.80 (−2.56 to 0.96)	0.370
Dyspnoea 12	18.00 (12.50–25.50) (n=56)	19.00 (9.00–24.00)	−0.57 (6.06)	18.50 (12.00–25.00) (n=54)	20.00 (12.00–26.00)	0.48 (5.36)	−1.23 (−3.31 to 0.85)	0.243
Medical research council breathlessness score	4.00 (3.00–4.00) (n=57)	4.00 (3.00–4.00)	−0.02 (0.81)	4.00 (3.00–4.00) (n=56)	4.00 (3.00–4.00)	−0.02 (0.56)	0.02 (−0.22 to 0.26)	0.865
Depression (PHQ-9)	7.00 (2.00–12.00) (n=57)	7.00 (3.00–14.00)	0.89 (4.44)	10.00 (3.00–14.00) (n=55)	10.00 (6.00–13.00)	0.73 (3.68)	−0.08 (−1.52 to 1.36)	0.913
Anxiety (GAD-7)	2.50 (0.00–7.50) (n=56)	1.00 (3.00–8.50)	0.71 (4.26)	4.00 (1.00–7.00) (n=55)	5.00 (1.00–8.00)	0.67 (3.29)	−0.05 (−1.45 to 1.35)	0.943
ABC score	75.63 (40.00–88.13) (n=57)	74.38 (41.25–86.88)	−0.89 (14.82)	73.13 (48.13–89.69) (n=55)	63.13 (38.75–86.88)	−7.35 18.62	6.15 (−0.079 to 12.37)	0.053
PROactive total score	55.00 (37.50–61.00) (n=49)	57.00 (40.50–63.50)	−0.19 (8.66)	51.25 (43.50–62.50) (n=40)	50.50 (43.75–59.50)	−0.40 (11.88)	−0.06 (−4.36 to 4.24)	0.978
PROactive amount score	50.00 (33.00–59.00) (n=49)	50.00 (33.00–63.00)	0.51 (7.95)	45.00 (33.00–54.00) (n=40)	45.00 (39.00–59.00)	1.27 (16.83)	−0.85 (−6.06 to 4.37)	0.747
PROactive difficulty score	56.00 (44.00–68.00) (n=54)	56.00 (44.00–70.00)	−0.41 (11.30)	56.00 (46.00–70.00) (n=54)	55.00 (44.00–70.00)	−2.02 (10.21)	1.41 (−2.67 to 5.50)	0.494
Daily step count	3678.25 (2190.60–5413.80) (n=51)	3315.80 (2022.00–5646.00)	248.96 (3274.24)	3575.60 (2174.00–4568.20 (n=41)	2844.00 (1284.40–5402.00)	−1060.33 (3810.53)	1225.77 (−79.16082 to 2530.702)	0.065

Data presented are median (IQR), or mean (SD, n (%) as stated.

ABC, Activities-specific Balance Confidence; CAT, chronic obstructive pulmonary disease assessment test; GAD-7, Generalised Anxiety Disorder-7 questionnaire; MHC, Mental Health Composite ; PHC, Physical Health Composite; PHQ-9, Patient Health Questionnaire (9 question); SF-36, 36-Item Short Form Health Survey.

**Table 4:** Responder analysis: number of participants experiencing a 10% improvement in the RAND SF-36 MHC score from baseline (imputation of missing follow-up data and complete case)

Experienced improvement >10% from baseline imputation of missing follow-up data)			
	Yes	No	
RAND SF-36 PHC score			p=0.025
SLH	16 (28.1%)	41 (71.9%)	57
Usual care	6 (11.1%)	48 (88.9%)	54
Total	22 (19.8%)	89 (80.2%)	111
RAND SF-36 MHC score			p=0.403
SLH	11 (19.3%)	46 (80.7%)	57
Usual care	14 (25.9%)	40 (74.1%)	54
Total	25 (22.5%)	86 (77.5%)	111
Experienced improvement >10% from baseline (complete case)			
RAND SF-36 PHC score			p=0.024
SLH	16 (32.0%)	34 (68.0%)	50
Usual care	6 (12.7%)	41 (87.2%)	47
Total	22 (22.7%)	75 (77.3%)	97
RAND SF-36 MHC score			p=0.381
SLH	11 (22.0%)	39 (78.0%)	50
Usual care	14 (29.8%%)	33 (70.2%)	47
Total	25 (25.8%)	72 (74.2%)	97

MHC, mental health composite; PHC, physical health composite; SF-36, 36-Item Short Form Health Survey; SLH, Singing for Lung Health.

Results for the complete case analysis and using baseline carried forward imputation provided very similar results to the ITT analysis (online supplemental table 1). The amount of missing data imputed is shown in online supplemental table 2.

A per-protocol analysis (SLH participants attending ≥9 sessions) also gave broadly similar results (online supplemental table 3). Of note, there was a slight attenuation of the SF-36 PHC p-value (regression coefficient 1.80 (95% CI −0.58 to 4.19); p=0.137), though the Physical Role Limitation SF-36 subscale was significantly different between groups (regression coefficient 12.16 (95% CI 0.10 to 24.22); p=0.048). Balance confidence (Activities-specific Balance Confidence (ABC) score) also demonstrated a clearer between-group difference (regression coefficient 9.54 (95% CI 0.96 to 18.11; p=0.030).

A small amount of questionnaire data was not collected or valid. Physical activity data at follow-up was only available for 33 and 31 participants in the intervention and UC arms, respectively, due to technical issues with the devices, including hardware and software failures, or participants not wearing them for sufficient time for valid data collection. The number of data points contributing to each outcome measure at baseline is shown in online supplemental table 2.

No serious intervention-related adverse events were reported. One participant attributed an increase in coughing to SLH participation. Adverse events were similar in the SLH compared with the UC study arms (16 (9.1%) vs 22 (37.9%), p=0.22).

## Discussion

The SHIELD Online study demonstrates that SLH, delivered using a telemedicine approach, is safe and well tolerated in COPD. Participation in once-weekly online SLH health sessions for 12 weeks improved the physical, but not mental, component of HRQoL. The overall group mean effect size was relatively small and did not meet the MCID for this test, but responder analysis found that people in the intervention were more likely to achieve a potentially clinically significant benefit compared with controls, with an NNT of 6.

Our findings are consistent with previous smaller RCTs and a recent systematic review of face-to-face SLH in people with COPD, which demonstrated an improvement in the SF-36 PHC^7 8 15 26^; however, the effect size in the present study was substantially lower than those studies. Delivery online, as opposed to in person, may have impacted effectiveness. Although the activity component of SLH participation has been demonstrated to increase metabolic demand,^27^ the extent to which people physically engage during online participation may have been less compared with being in person. In addition, the physical activity involved in actually attending the class venue is also lost. The background context of the COVID-19 pandemic is likely to have influenced its impact. Qualitative findings from pilot work at the start of the pandemic found participants with experience in both modalities preferred face-to-face to online.^13^ In a pilot RCT, which compared SLH to film club participation, the MHC improved in both groups, while the PHC only improved in the SLH group.^7^ The lack of MHC improvement in the current study could result from differences in psychosocial experiences between face-to-face and online sessions, or perhaps the background context of the COVID pandemic dominating experience of mental health well-being. Participants who had experience with both formats reported not feeling connected to the group online in the same way as in person.^13^ This may have reduced the effect on mental health compared with previous studies of face-to-face SLH.^7 8^

Physical activity was assessed using the cPACC PROactive measures (total, difficulty and amount) and daily step count. Between-group differences of these measures did not meet statistical significance. However, the context of the COVID-19 pandemic and related restrictions may have limited potential for change in these parameters; therefore, future research should include physical activity and performance assessment.

Data collection took place during the first 18 months of the COVID-19 pandemic, which is likely to have impacted the results. For example, it is notable that the between-group difference in PHC in the ITT analyses results from smaller declines in the SLH group than in the UC group, rather than larger improvements. This may suggest that SLH participation served to maintain, or limit negative impacts, to physical aspects of HRQoL, in the context of the COVID-19 pandemic and related public health measures. Public health measures limiting in-person interactions are likely to have had variable and dynamic effects on the impact of the intervention. Session leaders fed back informally that early in the pandemic, the online format was very well received as it facilitated social interaction in a safe way, but later in the study, the novelty of video conferencing had reduced and participants appeared less enthusiastic about the delivery format. Indeed, session attendance and withdrawal rates appeared to get worse in the final SLH groups.

### Methodological issues

A substantial number of people contacted declined to participate because they did not want to sing or did not like the online format of delivery (figure 3). Additionally, the requirement for digital literacy and a video conferencing capable device with an internet connection excluded others. Even after recruiting participants who were happy to try the activity, the attendance data demonstrated that participants attended the vast majority, or very few, of the sessions (figure 3). This may suggest that after the initial practical experience of SLH participation, it was relatively clear to participants whether they felt that participation was worthwhile and/or enjoyable—SLH can only benefit people who actually take part in it, which has implications for trial design. Although certain barriers exist regarding digital literacy and connectivity, it is worth noting that in the context of COVID-19 pandemic public health measures, the barriers to alternative approaches to delivery were far more prohibitive. Face-to-face SLH groups were legally prohibited during much of this period, and as such, the net impact of online delivery was an enabler. Similarly, online delivery overcomes many barriers that exist beyond pandemic-related restrictions, including travel and the availability of local SLH session provision. The relative impact of these barriers and facilitators in the future remains to be seen and is likely to vary over time and affect people differently. Providing the option for both face-to-face and online formats would most probably be the best approach, in a similar way to developments in the delivery of pulmonary rehabilitation. Within the trial population, there was a range of responses, with some participants reporting substantial benefits and others reporting none. In keeping with this, some attended the vast majority of sessions, while others quit early (figure 3). Further work could include improving the identification of those likely to respond and determining what factors might improve compliance. Providing more information about the content of the intervention before enrolling participants, might reduce the number of participants who quit early on.

Having to adapt the trial from face-to-face to online delivery resulted in additional methodological limitations. For example, the online version was relatively new when the trial began. It is possible that, with time, this format of delivery might be refined and made more effective. It is also possible that with increases in online activities in this age group more generally, acceptability and digital literacy may have improved, also moderating the experience and impact of the intervention. Adapting the format also meant that the 6MWT and SPPB were not included, which is unfortunate, as these measures would have provided an objective assessment of physical performance that may, or may not, have aligned with the changes in the PHC.

There is potential variation in the acceptability of SLH approaches in different cultural contexts beyond England and Wales. However, related interventions delivered in other cultural contexts, including Uganda and the Kyrgyz Republic^28^,^30^ suggest potential for contextual adaptation, though more research in this area is required.

Future research should aim to establish the impact of face-to-face SLH compared with usual care in larger, longer-term studies, and possibly comparing modes of delivery. Other research could explore different frequencies (eg, twice weekly, shorter/more intense and structured ‘homework’); SLH in other respiratory conditions and timing of intervention, for example, maintenance post-PR, in addition to PR, or in people who have not/will not do PR (possibly more potential for benefit) and longer-term respiratory choirs or similar weekly sessions. Future research using online SLH should consider how to improve MHC impacts to a level comparable with face-to-face delivery, or whether hybrid approaches that combine remote and face-to-face delivery might be a satisfactory compromise.

## Conclusion

Participation in 12 weeks of once-weekly online SLH by people with COPD is safe and results in small but statistically significant benefits in the physical, but not mental, component of HRQoL compared with UC alone. Considered in the context of research into SLH interventions for COPD, our results add more support to indications of physical benefits from participation. The results suggest that online SLH could be suggested to people with COPD as a potentially useful addition to usual care. Further large, longer-term studies are required to identify optimal methods for delivery, as well as the frequency, and timing of SLH programmes.

## Supplementary material

10.1136/bmjresp-2024-002365 online supplemental file 1

10.1136/bmjresp-2024-002365 Uncited online supplemental file 1

## Data Availability

Data are available upon reasonable request.
