# Supplementary material for: Singing for lung health in COPD: a multicentre randomised controlled trial of online delivery
Source: BMJ Open Respir Res. 2024 May 2;11(1):e002365. doi: 10.1136/bmjresp-2024-002365 (PMC11086531; doi:10.1136/bmjresp-2024-002365)
Supplement: online supplemental file 1 [file bmjresp-2024-002365supp001.pdf]

Online supplement

Singing for Lung Health in COPD: a multicentre randomised controlled trial of online delivery

Keir EJ Philip[1,2], Sara C Buttery[1,2], Sarah Bowen[3], Adam Lewis[4], Edmund Jeffery[2], Saeed M Alghamdi[1,2], Parris J Williams[1,2], Ali M Alasmari[1,2], Abdullah S Alsulayyim[1,2], Christopher M Orton[1,2], Francesca Conway[1,2], Ley Chan[1,2], Bavithra Vijayakumar[1,2], Anand Tana[1,2], James Tonkin[1,2], Alexis Perkins[1,2], Justin L Garner[1,2], Karthikan Srikanthan[1,2], Ahmed Sadaka[1,2], Matthew Pavitt[1,2], Winston Banya[2], Adam Lound[5], Sarah Elkin[6], Michael I Polkey[1,2], William Man[1,2], Keir Lewis[3], Phoene Cave[1], Daisy Fancourt[7], Nicholas S Hopkinson[1,2]

1) National Heart and Lung Institute, Imperial College London

2) Respiratory Medicine, Royal Brompton and Harefield Hospitals

3) Hywel Dda University Health Board (UHB), Carmarthen, Wales

4) Department of Health Sciences, Brunel University London

5) Patient Experience Research Centre, Imperial College London

6) Respiratory Medicine, Imperial College Healthcare NHS Trust

7) Department of Behavioural Science and Health, University College London, London, UK

Corresponding Author: Keir EJ Philip, email: k.philip@imperial.ac.uk

Supplementary Table 1: Complete Case analysis presented: ITT (no imputation)

	Singing for Lung Health online (SLH) (n=57)			Usual Care (UC) (n=58)				
Outcome Variable	Baseline	Follow-up	Change<br><br>(mean (SD))	Baseline	Follow-up	Change (mean (SD))	Regression coefficient (95% CI)	p-value
RAND SF-36 PHC Score	30.06 (22.24 -36.38) (n=50)	30.22 (23.27-35.50) (n=50)	-0.13 (5.27) (n=50)	29.03 (23.80 -37.86) (n=47)	27.22 (21.52-33.94) (n=47)	-2.10 (5.07) (n=47)	1.91 (0.03-3.79)	**0.046**
RAND SF-36 MHC Score	36.68 (32.05 -51.28) (n=50)	39.29 (29.73 -47.84) (n=50)	-1.10 (7.20) (n=50)	37.61 (27.76 -48.03) (n=47)	36.63 (28.06-43.49) (n=47)	-1.50 (8.12) (n=47)	0.91 (-1.97 - 3.79)	0.531
SF-36 Physical function	20.00 (5.00 -40.00)	22.50 (10.00 -45.00)	3.40 (14.55)	25.00 (10.00 -50.00)	25.00 (10.00 - 45.00)	0.11 (12.36)	2.50 (-2.69 - 7.69)	0.342
SF-36 Role limitation, physical	0.00 (0.00 - 50.00)	0.00 (0.00 - 50.00)	-2.50 (28.23)	0.00 (0.00 - 50.00)	0.00 (0.00 - 25.00)	-13.30 (32.91)	9.58 (-0.8821164 - 20.04)	0.072
SF-36 pain	56.50 (22.00 - 84.00)	51.50 (31.00 - 74.00)	-0.74 (25.68)	41.00 (22.00 - 62.00)	41.00 (22.00 - 62.00)	-3.43 (20.03)	4.51 (-3.93 - 12.95)	0.291
SF36 general health	25.00 (10.00 - 37.00)	22.00 (10.00 - 37.00)	-1.36 (13.03)	22.00 (15.00 - 42.00)	20.00 (10.00 - 37.00)	-2.87 (12.02)	1.35 (-3.46 - 6.16)	0.579
SF-36 energy	35.00 (20.00 - 50.00)	35.00 (20.00 - 50.00)	-0.30 (15.20)	25.00 (15.00 - 45.00)	30.00 (15.00 - 45.00)	0.11 (15.76)	1.81 (-3.79 - 7.41)	0.523
SF-36 Role limitation, emotional	66.67 (0.00 - 100.00)	66.67 (0.00 - 100.00)	2.67 (41.97)	33.33 (0.00 - 100.00)	33.33 (0.00 - 100.00)	-3.55 (47.77)	7.65 (-8.21 - 23.51)	0.341
SF-36 emotional wellbeing	66.00 (52.00 - 80.00)	66.00 (52.00 - 80.00)	-2.08 (13.81)	60.00 (52.00 - 84.00)	64.00 (40.00 - 76.00)	-3.23 (15.99)	2.13 (-3.44 - 7.71)	0.449
SF-36 social functioning	50.00 (25.00 - 75.00)	50.00 (25.00 - 62.50)	-0.30 (15.20)	50.00 (25.00 - 75.00)	50.00 (25.00 - 75.00)	0.11 (15.76)	-0.30 (-6.40 - 5.79)	0.922
CAT score	25.00 (18.00 - 28.00) (n=50)	24.00 (16.00 - 29.00) n=50)	-0.20 (4.74) (n=50)	24.00 (19.00 - 29.00) (n=49)	24.00 (16.00 - 31.00) (n=49)	0.73 (5.28) (n=49)	-0.91 (-2.89 - 1.06)	0.362
Dyspnoea 12	18.50 (10.50 - 26.00) (n=48)	19.00 (8.00 - 24.50) (n=48)	-0.67 (6.55) (n=48)	18.00 (11.00 - 24.00) (n=48)	19.50 (12.00 - 24.00) (n=48)	0.54 (5.69) (n=48)	-1.27 (-3.65 - 1.10)	0.290
MRC Dyspnoea score	4.00 (3.00 - 4.50) (n=48)	4.00 (3.00 - 4.00) (n=48)	-0.02 (0.89) (n=48)	4.00 (3.00 - 4.00) (n=47)	4.00 (3.00 - 4.00) (n=47)	-0.02 (0.61) (n=47)	-0.0040 (-(0.29 - 0.28)	0.978

Depression (PHQ-9)	6.50 (2.00 - 12.00) (n=50)	8.00 (3.00 - 14.00) (n=50)	1.02 (4.73) (n=50)	10.00 (3.00 - 14.00) (n=50)	10.00 (6.00 - 13.00) (n=50)	0.80 (3.85) (n=50)	-0.12 (-1.71 - 1.47)	0.880
Anxiety (GAD-7)	2.00 (0.00 - 6.00) (n=47)	3.00 (1.00 - 8.00) (n=47)	0.85 (4.65) (n=47)	4.00 (1.00 - 8.00) (n=50)	5.00 (1.00 - 8.00) (n=50)	0.74 (3.45) (n=50)	-0.094 (-1.70 - 1.51)	0.907
ABC score	77.19 (45.94 - 88.44) (n=48)	75.78 (44.38 - 87.50) (n=48)	-1.06 (16.17) (n=48)	75.00 (49.38 -90.00) (n=50)	65.31 (38.75 - 87.50) (n=50)	-8.09 (19.40)	6.89 (-0.20 - 13.98)	0.057
PROactive Total Score	54.25 (37.50 - 61.00) (n=34)	57.00 (40.50 - 64.00) (n=34)	-0.28 (10.45) (n=34)	51.50 (42.50 -62.50) (n=31)	51.00 (42.50 -59.50) (n=31)	-0.52 - (13.54) (n=31)	-.0044 (-5.88 - 5.87)	0.999
PROactive Amount score	45.00 (33.00 - 59.00) (n=33)	45.00 (33.00 - 63.00) (n=33)	0.76 (9.73) (n=33)	45.00 (33.00 - 54.00) (n=31)	45.00 (33.00 - 59.00) (n=31)	1.65 (19.17) (n=31)	-0.89 (-8.07 - 6.29)	0.804
PROactive Difficulty score	56.00 (46.00 - 66.00) (n=45)	56.00 (44.00 - 70.00) (n=45)	-0.49 (12.40) (n=45)	56.00 (48.00 - 70.00) (n=47)	55.00 (46.00 - 70.00) (n=47)	-2.32 (10.92) (n=47)	1.52 (-3.29 to 6.33)	0.532
Daily step count	2833.60 (1917.90 – 5347.90) (n=40)	3066.50 (1934.30 - 5203.10)	317.43 (3704.34)	3341.10 (2174.00 - 4568.20) (n=38)	2825.20 (1284.40 - 5782.20)	-1144.04 (3949.57)	1056.965 (-451.0619 - 2564.991)	0.167

Supplementary Table 2: Completeness of outcome measure data at baseline

	Intervention arm (SLH) (n=57)	Usual Care (UC) (n=58)
**Outcome measures at baseline**		
RAND SF-36 PHC Score	57 (100%)	54 (93.1%)
RAND SF-36 MHC Score	57 (100%)	54 (93.1%)
CAT score	57 (100%)	54 (93.1%)
Dyspnoea 12	56 (98.2%)	54 (93.1%)
MRC Dyspnoea score	57 (100%)	58 (100%)
Depression (PHQ-9)	57 (100%)	55 (94.8%)
Anxiety (GAD-7)	56 (98.2%)	55 (94.8%)
ABC score	57 (100)	55 (94.8%)
PROactive Total Score	49 (86.0%)	40 (70.0%)
PROactive Amount score	49 (86.0%)	40 (70.0%)
PROactive Difficulty score	54 (94.7%)	54 (93.1%)
Daily step count	51 (89.5%)	41 (70.1%)

Note: 4 participants (4012, 4013, 4014, 4030), from UC arm where allocated but did not provide valid SF-36 data at BL. Similarly, 3 participants (3919, 4004, 4008) completed study but did not provide valid SF36 FU data. 2 SLH participants stopped attending sessions due to technical difficulties with internet/computer stopped working. However, they completed follow up data collection, and did not withdraw from the study.

Supplementary Table 3: Complete Case, per protocol (min. 9 session attendance in intervention arm)

	Singing for Lung Health online (SLH) (n=29)			Usual Care (UC) (n=58)				
Outcome Variable	Baseline	Follow-up	Change (mean (SD))	Baseline	Follow-up	Change (mean (SD))	Regression coefficient (95% CI)**	p-value
RAND SF-36 PHC Score	33.26 (27.84 - 40.37) (n=29)	32.92 (26.77 - 39.07) (n=29)	-1.13 (6.01)	29.03 (23.80 - 37.86) (n=47)	27.22 (21.52 - 33.94) (n=47)	-2.10 (5.07)	1.80 (-0.58 - 4.19)	0.137
RAND SF-36 MHC Score	39.62 (35.38 - 51.78) (n=29)	39.76 (31.54 - 47.84) (n=29)	-1.58 (6.19)	37.61 (27.76 - 48.03) (n=47)	36.63 (28.06 - 43.49) (n=47)	-1.50 (8.12)	0.997 (-2.29 - 4.29)	0.548
SF-36 Physical function	25.00 (15.00 - 40.00)	30.00 (10.00 - 55.00)	1.21 (13.87)	25.00 (10.00 - 50.00)	25.00 (10.00 - 45.00)	0.11 (12.36)	1.72 (-4.11 - 7.55)	0.558
SF-36 Role limitation, physical	25.00 (0.00 - 50.00)	0.00 (0.00 - 50.00)	-2.59 (27.01)	0.00 (0.00 - 50.00)	0.00 (0.00 - 25.00)	-13.30 (32.91)	12.16 (0.10 - 24.22)	**0.048**
SF-36 pain	74.00 (41.00 - 100.00)	62.00 (41.00 - 84.00)	-4.55 (31.28)	41.00 (22.00 - 62.00)	41.00 (22.00 - 62.00)	-3.43 (20.03)	6.54 (-4.43 - 17.52)	0.238
SF36 general health	30.00 (20.00 - 42.00)	25.00 (15.00 - 40.00)	-3.48 (14.70)	22.00 (15.00 - 42.00)	20.00 (10.00 - 37.00)	-2.87 (12.02)	0.38 (-5.46 - 6.22)	0.898
SF-36 energy	45.00 (25.00 - 55.00)	40.00 (25.00 - 50.00)	-1.55 (15.47)	25.00 (15.00 - 45.00)	30.00 (15.00 - 45.00)	0.11 (15.76)	2.35 (-4.21 - 8.92)	0.477
SF-36 Role limitation, emotional	100.00 (33.33 - 100.00)	100.00 (0.00 - 100.00)	-2.30 (34.42)	33.33 (0.00 - 100.00)	33.33 (0.00 - 100.00)	-3.55 (47.77)	7.45 (-11.08 - 25.98)	0.426
SF-36 emotional wellbeing	64.00 (60.00 - 80.00)	68.00 (56.00 - 84.00)	-0.28 (9.85)	60.00 (52.00 - 84.00)	64.00 (40.00 - 76.00)	-3.23 (15.99)	4.40 (-1.60 - 10.40)	0.148
SF-36 social functioning	62.50 (37.50 - 87.50)	50.00 (25.00 - 75.00)	-1.55 (15.47)	50.00 (25.00 - 75.00)	50.00 (25.00 - 75.00)	0.11 (15.76)	-0.86 (-8.09 - 6.37)	0.814
CAT score	24.00 (17.00 - 27.00 (n=29)	14.00 (20.00 - 29.00) (n=29)	-0.24 (4.73)	24.00 (19.00 -29.00) (n=49)	24.00 (16.00 - 31.00)	0.73 (5.28)	-1.20 (-3.58 - 1.18)	0.319
Dyspnoea 12	17.00 (8.00 - 26.00) (n=29)	18.00 (6.00 -24.00) (n=29)	-0.76 (6.09)	18.00 (11.00 - 24.00) (n=48)	19.50 (12.00 - 24.00) (n=48)	0.54 (5.69)	-1.66 (-4.29 - 0.98)	0.215

MRC Dyspnoea score	4.00 (3.00 -4.00) (n=28)	3.50 (3.00 -4.00) (n=28)	-0.04 (0.79)	4.00 (3.00 -4.00) (n=47)	4.00 (3.00 - 4.00) (n=47)	-0.02 (0.61)	-0.07 (-0.37 - 0.23)	0.639
Depression (PHQ-9)	5.00 (2.00 - 10.00) (n=29)	7.00 (4.00 - 13.00) (n=29)	1.76 (4.52)	10.00 (3.00- 14.00) (n=50)	10.00 (6.00 - 13.00) (n=50)	0.80 (3.85)	0.28 (-1.50 - 2.05)	0.758
Anxiety (GAD-7)	2.00 (0.00 - 6.00) (n=29)	3.00 (1.00 - 8.00) (n=29)	0.55 (4.32)	4.00 (1.00 - 8.00) (n=50)	5.00 (1.00 - 8.00) (n=50)	0.74 (3.45)	-0.53 (-2.26 - 1.20)	0.540
ABC score	83.44 (73.44 - 91.56) (n=28)	84.06 (63.44 - 91.09)	-0.07 (17.06)	75.00 (49.38 - 90.00) (n=50)	65.31 (38.75 - 87.50)	-8.09 (19.40)	9.54 (0.96 - 18.11)	**0.030**
PROactive Total Score	55.00 (37.50 - 66.50) (n=19)	57.00 (41.50 - 64.00) (n=19)	0.61 (5.78)	51.50 (42.50 - 62.50) (n=31)	51.00 (42.50 - 59.50) (n=31)	-0.52 (13.54)	1.35 (-4.84 - 7.53)	0.664
PROactive Amount score	50.00 (33.00 - 63.00) (n=19)	50.00 (33.00 - 63.00) (n=19)	2.53 (11.52)	45.00 (33.00 - 54.00) (n=31)	45.00 (33.00 - 59.00) (n=31)	1.65 (19.17)	1.77 (-7.49 - 11.03)	0.703
PROactive Difficulty score	58.00 (51.00 - 70.00) (n=25)	56.00 (44.00 - 68.00) (n=25)	-1.92 (8.90)	56.00 (48.00 - 70.00) (n=47)	55.00 (46.00 - 70.00) (n=47)	-2.32 (10.92)	0.58 (-4.35 - 5.51)	0.816
Daily step count	3975.00 (2240.40 - 5413.80) (n=23)	3673.40 (2070.80 - 5646.00) (n=23)	-30.37 (2326.35)	3341.10 (2174.00 - 4568.20) (n=38)	2825.20 (1284.40 - 5782.20) (n=38)	-1144.04 (3949.57)	988.69 (-310.1301 - 2287.51)	0.133
